# Evaluation of a self-management patient education program for patients with fibromyalgia syndrome: study protocol of a cluster randomized controlled trial

**DOI:** 10.1186/s12891-016-0903-4

**Published:** 2016-02-03

**Authors:** Gunda Musekamp, Christian Gerlich, Inge Ehlebracht-König, Hermann Faller, Andrea Reusch

**Affiliations:** Department of Medical Psychology and Psychotherapy, Medical Sociology and Rehabilitation Sciences, University of Würzburg, Klinikstr. 3, D-97070 Würzburg, Germany; Rehabilitation Center Bad Eilsen, Brunnenpromenade 2, D-31707 Bad Eilsen, Germany

**Keywords:** Fibromyalgia syndrome, Patient education, Self-management, Evaluation, Cluster-RCT, Rheumatology, Rehabilitation

## Abstract

**Background:**

Fibromyalgia syndrome (FMS) is a complex chronic condition that makes high demands on patients’ self-management skills. Thus, patient education is considered an important component of multimodal therapy, although evidence regarding its effectiveness is scarce. The main objective of this study is to assess the effectiveness of an advanced self-management patient education program for patients with FMS as compared to usual care in the context of inpatient rehabilitation.

**Methods/Design:**

We conducted a multicenter cluster randomized controlled trial in 3 rehabilitation clinics. Clusters are groups of patients with FMS consecutively recruited within one week after admission. Patients of the intervention group receive the advanced multidisciplinary self-management patient education program (considering new knowledge on FMS, with a focus on transfer into everyday life), whereas patients in the control group receive standard patient education programs including information on FMS and coping with pain. A total of 566 patients are assessed at admission, at discharge and after 6 and 12 months, using patient reported questionnaires. Primary outcomes are patients’ disease- and treatment-specific knowledge at discharge and self-management skills after 6 months. Secondary outcomes include satisfaction, attitudes and coping competences, health-promoting behavior, psychological distress, health impairment and participation. Treatment effects between groups are evaluated using multilevel regression analysis adjusting for baseline values.

**Discussion:**

The study evaluates the effectiveness of a self-management patient education program for patients with FMS in the context of inpatient rehabilitation in a cluster randomized trial. Study results will show whether self-management patient education is beneficial for this group of patients.

**Trial registration:**

German Clinical Trials Register, DRKS00008782, Registered 8 July 2015

## Background

This is a study protocol of a cluster randomized trial based on CONSORT and SPIRIT recommendations [1, 2].

The fibromyalgia syndrome (FMS) is characterized by chronic widespread pain and additional symptoms as fatigue, unrefreshed sleep, cognitive symptoms and various somatic symptoms [3]. About 2–7 % of the general population are affected by FMS, with a higher prevalence in women [4–6]. The disorder causes substantial burden for the individual [7] and high socioeconomic costs through increased healthcare utilization [8]. A study of a German health insurance company estimated total yearly costs to 4.331 €, thereof 3602 € direct costs [9]. Another study calculated adjusted yearly direct costs of $2417 and indirect costs of $10.001 in Germany [10]. In an US-sample, mean healthcare costs for FMS patients were three times higher than for other patients [11]. According to FMS guidelines [12–14], treatment should be multidisciplinary and combine different pharmacologic and non-pharmacologic therapies, with self-management patient education as one recommended component. Research indicates that multicomponent therapy including psychological and exercise therapy has positive effects on key symptoms of FMS, self-efficacy and physical fitness [15]. Information about the relative effectiveness of different treatment components is scarce [16]. Meta-analytical results show small but robust positive effects of psychological treatments (including patient education) for fibromyalgia [17]. There is limited evidence regarding the effectiveness of self-management patient education for patients with FMS. Comparison of studies is difficult because of different contents and formats of education programs and evaluation of multimodal programs with different components [18–33]. Moreover, many studies use some form of education as control (not as intervention) condition or do not assess long-term effectiveness of self-management education [34]. Further research is needed to determine the independent effects of patient education [35].

An existing German patient education program for patients with FMS in the medical rehabilitation system [36] proved its worth over years, but there was a need to revise it. It did not consider up-to-date knowledge about FMS, especially the new national guideline [12], had potential for didactical improvements and should be advanced with regard to patients’ needs [37]. Furthermore, its effectiveness has not been evaluated so far. Therefore, based on this program, an advanced self-management patient education program was developed. This new interactive, didactically revised program aims to promote a biopsychosocial understanding and self-management of the disease. It considers current knowledge on FMS and is consistent with the current national guideline [12]. It has a focus on the transfer of pain and stress management and physical activity into everyday life. Although content and structure are similar to the prior program, it includes more practical exercises and emphasizes transfer into everyday life through action planning.

The objective of this cluster randomized controlled study is to evaluate this advanced self-management patient education program for patients with fibromyalgia syndrome [38] in inpatient rehabilitation. The main research questions are the short-, intermediate- and long-term effectiveness of the self-management program compared to usual care, in this case an active control (standard patient education programs). We hypothesize that the program is superior to usual care regarding both disease- and treatment-specific knowledge at discharge of rehabilitation and self-management competence 6 months after rehabilitation (primary outcomes). Additionally, we expect superior effectiveness regarding satisfaction with the program, attitudes towards the illness and coping competences, health-promoting behavior, psychological distress, health impairment and participation (secondary outcomes). The role of potential moderator variables (e.g. age, psychological co-morbidity, duration of disease) is examined in an exploratory manner.

## Methods/Design

### Study design and data collection

**Fig. 1:**
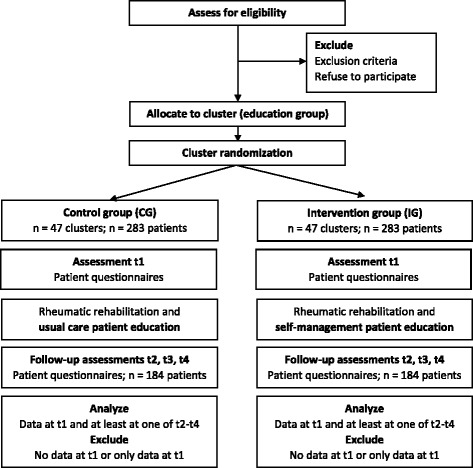
Study protocol diagram of data collection process

This study is a multicenter cluster randomized controlled trial (RCT) conducted in three German inpatient rehabilitation clinics. Clusters are groups of patients with FMS consecutively recruited within one week after admission to the clinic. The superiority trial has two arms. Patients of the intervention group (IG) receive the advanced self-management patient education program. Patients of the control group (CG) receive standard patient education programs including information on FMS and coping with pain (usual care). Data are collected at admission (t1), discharge (t2) and after 6 (t3) and 12 months (t4) with standardized and self-developed patient-reported questionnaires. Additionally, medical data are collected during rehabilitation. Cluster randomization was chosen for practical reasons (to have groups that are sufficiently large), for blinding patients as to intervention conditions and to prevent contamination between interventions. Clusters of patients are randomly assigned to the two intervention conditions in a 1:1 ratio. For t1 and t2, patients receive the questionnaire in the inpatient rehabilitation clinic. For t3 and t4, patients receive the questionnaire by mail and are reminded with a second letter if necessary. Figure 1 shows the study protocol diagram.

### Ethical aspects

This study conforms to the principles of the Declaration of Helsinki. It was approved by the Ethics Committee of the Faculty of Medicine, University of Würzburg, on April 22, 2014 (reference number: 46/14). Participants are informed about the study in the initial consultation and additionally are provided an information sheet. Participation is voluntary and based on written informed consent. Patients are informed about their right to refuse participation in the study or withdraw consent to participate without consequences for their treatment. They are also informed about data protection through collecting data using code numbers for each patient.

### Participants

Considered for inclusion are adult patients in inpatient rehabilitation clinics with the main diagnosis fibromyalgia syndrome (ICD-10: M79.7). Exclusion criteria are insufficient German language ability, severe psychiatric comorbidity which leads to inability to participate in an educational group, cognitive impairment or not corrected severe visual or hearing impairment. Patients with FMS are admitted to the clinics every two weeks. All patients admitted during this period of one week are eligible to build a cluster, with a minimum of 3 participants per group.

### Intervention

Inpatient rehabilitation in Germany for patients with FMS is multimodal and includes medical treatment, exercise therapy, health education, psychological support, relaxation and social counseling over a period of three (for severe cases sometimes four) weeks. This trial compares a self-management patient education group program to standard patient education programs (usual care), both within inpatient rheumatological rehabilitation centers.

Intervention group (IG). Participants in the intervention group attend a disease-specific self-management patient education program ([38], see Table 1). It consists of 6 sessions of 90 min each and one (optional) preparing session, all delivered in small groups (12 participants maximum) of closed format. The program is manual-based and led by physicians, psychologists and physiotherapists, respectively. Methods are interactive, with patients actively involved and different didactic methods used (interactive short lectures, group discussion, practice, individual work). Materials include presentations, flipchart, work sheets and patient information sheets. Contents of the sessions include fibromyalgia-related information (e.g. symptoms, diagnosis, course of disease, causes and influencing factors, frequency), information about treatment (e.g. treatment options, medication, physical therapy, psychotherapy, risks and benefits of alternative therapies), coping with pain and stress as well as promotion of sustained physical activity. There is a focus on both self-management and transfer into everyday life through action planning. Evaluations by patients performed during formative evaluation showed a high satisfaction with the program [38]. All trainers are experienced with patients with FMS. Additionally, the trainers receive a 1.5 day train-the-trainer course held by project staff and a psychologist with experience in FMS-education.

**Table 1:** Structure of the advanced self-management patient education program for patients with fibromyalgia syndrome

Session		Trainer
0	Introduction, questions and expectations (optional)	Psychologist
1	The fibromyalgia syndrome – What does that mean? (symptoms, diagnosis, course, causes and influencing factors, frequency, communication with health professionals)	Physician
2	How is the fibromyalgia syndrome treated? (treatment options, medication, physical therapy, psychotherapy, risks and benefits of alternative therapies)	Physician
3	Dealing with pain (acute vs. chronic pain, development and sustainment of pain, consequences, relationships to depression and anxiety, coping with pain)	Psychologist
4	Everything hurts! – Thus, why moving? (possibilities of physical activity, consequences, physical exercises, important principals of training, action and coping planning)	Physiotherapist
5	Everything grows over my head! – Ways out of stress (types of stressors, development of stress, stress reaction and consequences, relationship to pain, coping with stress, exercises)	Psychologist
6	Managing daily life (explaining the condition to others, goals for daily life, checking the action plans, improving sleep quality, personal conclusion)	Psychologist

Control group (CG). Control condition is the treatment as usual including education as delivered in the respective clinic. In one cooperating clinic, usual care is the previously practiced, interactive patient education for fibromyalgia syndrome with 5 sessions (90 min each; content: illness and treatment information, coping with pain and stress, exercise therapy). Trainers are a psychologist, a physician, a physiotherapist and an occupational therapist or nutritionist. In the second cooperating clinic, patients participate in a coping with pain group with 4 sessions (90 min each; content: information about pain, coping with pain, transfer into daily life) led by a psychologist, physiotherapist and physician and receive a flyer about the fibromyalgia syndrome. In the third cooperating clinic, patients are informed by 3 physician-delivered lectures (60 min each) about chronic pain, fibromyalgia syndrome and stress and may participate in educational group programs for pain or stress coping (each with 4 sessions of 45 min each, led by psychologists) or autogenic training, as indicated. There are differences between the three control conditions, but they also have common characteristics. They all include information about FMS and coping with pain. In contrast to the intervention condition, control conditions do not explicitly consider the up-to-date knowledge about FMS, the new guideline [12] and newly recorded needs of patients with FMS [38]. They are partly less interactive and less self-management-oriented as they do not include action and coping planning and do not have such a strong emphasis on transfer into everyday life.

### Outcomes and Measures

In this study, the effects of the treatment conditions are assessed at the individual level using patient-reported outcomes. Different proximal and distal outcomes at different measurement points are used for evaluation [39]. The primary outcome is disease- and treatment-specific knowledge at discharge of inpatient rehabilitation and patients’ subjective self-management competence 6 months after rehabilitation. Secondary outcomes include treatment satisfaction, attitudes towards the illness and coping competences, health-promoting behavior, psychological distress, health impairment and participation. For measuring knowledge, a new measure was developed based on the content of the patient education program (the same information is also given in the control condition). Acceptance of the disease and communication about the disease with others are also measured with a self-developed questionnaire. Both were pre-tested in a sample of n = 91 patients with fibromyalgia, items were selected due to difficulty and comprehensibility (knowledge) or results from factor analysis (acceptance and communication). The other outcomes are assessed by standardized, validated measures. Detailed information concerning the outcomes and questionnaires are shown in Table 2. It will take about 45 min for the patient to fill out the questionnaires at each measurement point.

**Table 2:** Outcomes, measures, assessment

Outcome	Measure	Assessment			
		t1	t2	t3	t4
*Primary outcomes*					
Disease- and treatment-specific knowledge	Self-developed questionnaire	x	x	-	x
Subjective self-management competence	Health education impact questionnaire heiQ, German version [40], subscales Skill and technique acquisition, Self-monitoring and insight	x	x	x	x
*Secondary outcomes*					
Treatment satisfaction	Patient education satisfaction questionnaire [45]	-	x	x	x
Attitudes and coping competences:					
Pain-related controllability	Rheumatology Attitudes Index, German version [46]	x	x	x	x
Acceptance of the disease and communication with others	Self-developed questionnaire	x	x	x	x
Behavior determinants for physical activity and relaxation	HAPA-scales [47, 48]	x	x	-	-
Behavior:					
Health-promoting behavior, active lifestyle	Godin Leisure-Time Exercise Questionnaire [49], German modified version	x	-	x	x
	Relaxation index [50]	x	-	x	x
	Health education impact questionnaire heiQ, German version, subscales Active engagement in life, Health-promoting activities	x	-	x	x
Interaction in the health system	Health education impact questionnaire heiQ, German version, subscale Health service navigation	x	-	x	x
Health impairment	Fibromyalgia Impact Questionnaire German version, without the physical functioning subscale, FIQ-G [51, 52]	x	x	x	x
Psychological distress	Short form of the Patient Health Questionnaire PHQ-4 [53]	x	x	x	x
Participation	Fibromyalgia Participation Questionnaire FPQ [54]	x	-	x	x

Additionally, potential moderator variables as sociodemographic parameters, duration of disease and social support (subscale social integration and support of the Health Education Impact Questionnaire heiQ [40]) are assessed as reported by the patients. Medical data as diagnoses, medication, ACR-criteria (diagnostic criteria of the American College of Rheumatology [3]) or working ability are provided by the attending physicians.

### Sample size

The needed sample size was calculated based on expected effect size, power and design effect (intracluster correlation, expected average cluster size, [41]). This calculation comprises some uncertainties as size of ICC, actual cluster size, effect sizes and dropout rates. There are no previous studies in similar contexts with similar interventions and outcomes which enable exact estimations. We assumed small to medium effects (*d* = 0.3) in the primary outcome due to the comparison with a control group that also gets a comprehensive treatment. Thus, setting alpha risk to 0.05 and power to 0.8, a sample of 175 per group is needed for two-tailed tests. Assuming an intraclass correlation coefficient (ICC) of 0.01 and an average cluster size of 6, the resulting design effect is 1.05, resulting in a needed sample size of 184 per group. Thus, 566 participants have to be recruited, based on an estimated maximum dropout rate of 35 % during follow-up. Because power to detect smaller effects between groups may be too low, effect sizes will be reported.

### Randomization, allocation concealment and blinding

External cluster-randomization is performed by the research institute (central randomization) using a computer-generated list of random numbers, ensuring a 1:1 ratio for both treatment conditions. FMS patients are recruited at admission to inpatient rehabilitation by study assistants in the clinics and allocated to the next education group (cluster). A new treatment condition starts every two weeks. Allocation concealment will be attained by providing information about allocation (by telephone or email) only after a cluster has been recruited. Patients are blinded regarding the allocated study arm. It is not possible to blind staff in the rehabilitation clinics, especially those performing the educational interventions.

### Statistical methods

All analyses are performed with SPSS for Windows. After exploration of missing patterns, missing data in accordance with missing at random assumptions will be imputed by a multiple imputation procedure. T-tests for independent variables are used for nonresponder- and dropout-analyses. The evaluation of primary and secondary endpoints is carried out according to the intention-to-treat-principle. Before analyzing the combined data of the three clinics we will confirm that 'clinic' is not a moderator variable. Treatment effects between groups will be evaluated separately for each follow-up time point using multilevel regression analysis adjusting for baseline values [41–43]. Treatment group is included in the models as fixed effect and individuals are nested in clusters (education groups) as random effects. Report of outcomes includes ICCs, statistical significance (*p* < 0.05, 2-sided) and effect sizes [44] for differences between groups. For exploratory moderator analyses, potential moderator variables are included as an additional fixed factor or covariate, respectively.

## Discussion

Fibromyalgia syndrome is an impairing condition and coping with pain and other sequelae is an important issue in its treatment. Thus, self-management patient education aiming at coping with the condition in everyday life may be an important component of treatment. Effectiveness of such programs within a multimodal treatment is still unclear, however. This study will contribute to evidence in that it will show whether an advanced patient education program implemented in inpatient rehabilitation centers is effective regarding disease- and treatment specific knowledge, subjective self-management competences and other outcomes. Therefore, it is compared to standard patient education programs (usual care). Outcomes are assessed not only in the short term, but also over a period of one year after rehabilitation. Multilevel analyses ensures the consideration of the hierarchical data structure, preventing misinterpretations due to dependency of individuals within clusters. Cluster randomization blinds patients and prevents contamination of the treatment. If the intervention proves to be effective, it may be implemented in other clinics and recommended to help satisfy the high need for disease-related information and self-management support of patients with FMS [38].
